# A tRF‐5a fragment that regulates radiation resistance of colorectal cancer cells by targeting *MKNK1*

**DOI:** 10.1111/jcmm.17982

**Published:** 2023-10-21

**Authors:** Tianyi Huang, Chujia Chen, Juan Du, Zhen Zheng, Shuang Ye, Shuai Fang, Kaitai Liu

**Affiliations:** ^1^ Department of Radiation Oncology The Affiliated Lihuili Hospital of Ningbo University Ningbo Zhejiang China; ^2^ Health Science Center Ningbo University Ningbo Zhejiang China; ^3^ Department of Thoracic Surgery The Affiliated Hospital of Medical School of Ningbo University Ningbo Zhejiang China

**Keywords:** colorectal cancer, MNNK1, radiation resistance, tRF‐16‐7X9PN5D, tRNA‐derived fragments

## Abstract

Radiotherapy serves as a crucial strategy in the treatment of colorectal cancer (CRC). However, its efficacy is often hindered by the challenge of radiation resistance. Although the literature suggests that some tRNA‐derived small RNAs (tsRNAs) are associated with various cancers, studies reporting the relationship of tsRNAs with cancer cell radiosensitivity have not been published yet. In our study, we utilized tsRNAs sequencing to predict differentially expressed tsRNAs in two CRC cells and their radioresistant cells, and 10 tsRNAs with significant differences in expression were validated by qPCR. The target genes of tRF‐16‐7X9PN5D were predicted and verified by the bioinformatics, dual‐luciferase reporter gene assay and western blotting analyses. Wound healing, colony formation, transwell invasion and CCK‐8 assays were performed to detect the effects of tRF‐16‐7X9PN5D on cell function and radiosensitivity. Western blotting evaluated the relationship between tRF‐16‐7X9PN5D and the MKNK‐eIF4E axis. Our findings demonstrated that tRF‐16‐7X9PN5D expression was substantially downregulated in radioresistant CRC cells. Furthermore, tRF‐16‐7X9PN5D could promote CRC cells' ability to proliferate, migrate, invade and obtain radiation resistance by targeting *MKNK1*. Finally, tRF‐16‐7X9PN5D could regulate eIF4E phosphorylation via *MKNK1*. This investigation indicated that tRF‐16‐7X9PN5D has an essential regulatory role in the radiation resistance of CRC by directly targeting *MKNK1*, and may be a new pathway for regulating the CRC radiosensitivity.

## 1 INTRODUCTION

Colorectal cancer (CRC) is the cancer of both colon and rectal cancer and is currently ranked as the third most frequent malignant tumour worldwide.^1^ The latest numbers provided by the International Agency for Research on Cancer (IARC) suggest that over 1.9 million individuals are diagnosed with CRC yearly, and 935,000 pass away.^1^ Unlike other cancers, CRC is not attributed to a single high‐risk factor but rather the result of multiple risk factors working together.^2^ Therefore, most CRC cases are still sporadic and develop slowly via the adenoma‐carcinoma sequence over several years.^2^ Currently, CRC treatment options include surgery, adjuvant radiotherapy and chemotherapy, immunotherapy and targeted therapy.^3^, ^4^ Among them, radiotherapy is an essential comprehensive treatment for locally advanced rectal cancer, and it substantially improves patients' quality of life and prognosis.^5^ However, due to the different sensitivities of various tumours and radiotherapy stages, radiation resistance is still one of the main factors limiting radiotherapy efficacy.^6^ Therefore, comprehensive research on the molecular mechanisms affecting CRC radioresistance is of great clinical significance.

Recently, multiple studies have indicated that abnormal cell signalling pathways activation, cell apoptosis escape, cell cycle checkpoint activation and abnormal expression of some microRNAs are closely related to radiation resistance.^7^ Small noncoding RNAs (sncRNAs) comprise various types of RNAs, of these tRNA‐derived small RNA (tsRNA) is among the most abundant and ancient sncRNAs, accounting for 4%–10% of all cellular RNAs, generated through tRNA cleavage.^8^, ^9^, ^10^ According to different cleavage positions and lengths, tsRNAs can be of two main types: tRNA‐derived fragments (tRFs) and tRNA halves (tRHs or tiRNAs).^11^, ^12^ Increasing evidence has revealed that tsRNAs dysregulation can affect tumour incidence and development via mechanisms, including transcriptional inhibition, posttranscriptional regulation, and protein synthesis regulation.^13^, ^14^, ^15^ They are also crucial for regulating cell activity and signalling pathways.^16^, ^17^ For example, tRF3008A overexpression inhibits CRC cell's ability to proliferate, migrate and invade by suppressing endogenous *FOXK1*, whereas tRF‐17‐79MP9PP targets *THBS1* to repress the TGFb1/Smad3 signalling pathway in breast cancer cells.^18^, ^19^ Additionally, tsRNAs have potential therapeutic, diagnostic and prognostic value in various cancers such as that colorectal,^18^ pancreas,^20^ breast,^21^ liver^22^ and ovaries.^23^ However, no studies currently report the relationship between tsRNA and cancer cell radiation sensitivity.

MAPK interacting serine/threonine kinase‐1 (*MKNK1*) is a member of the MAPK subfamily that acts as a critical regulatory factor in the MAPK signalling pathway.^24^, ^25^ In recent years, numerous new studies have also confirmed the significant role of the MAPK signalling pathway in human diseases.^26^, ^27^, ^28^ Its encoded protein MKNK1, as a type of tyrosine/threonine kinase, can phosphorylate multiple substrates, including apoptosis and growth factor receptors, transcription factors, ribosomal proteins and other kinases.^29^, ^30^ The literature indicates that *MKNK1* has potential clinical applications for various malignant tumours, such as CRC,^31^ breast cancer^32^ and glioblastoma.^33^ However, research on the sncRNA modulation of *MKNK1* is limited, and only a few studies reveal that miRNAs can target *MKNK1* to affect cancer progression.^34^ There is no research revealing the relationship between tsRNAs and *MKNK1* yet.

This investigation was the first to discover that tRF‐16‐7X9PN5D is markedly linked with the radiation sensitivity of CRC cells. Inhibition of tRF‐16‐7X9PN5D promotes CRC cells' ability to proliferate, migrate and invade by directly targeting *MKNK1* and may exert its molecular function through the MKNK‐ eIF4E axis.

## 2 MATERIALS AND METHODS

### 2.1 Cell lines and cell culture

Human CRC cell lines (SW620 and HCT116) were provided by Zhejiang Ruyao Biotechnology Co., Ltd. The cells were propagated in DMEM media (Gibco) augmented with 10% fetal bovine serum (Gibco) at 37°C in a 5% CO_2_ humidified incubator. To obtain radiation‐resistant cells (SW620R and HCT116R), ionising radiation was applied to the cells using a graded increase method.^35^ Cultured wild‐type SW620 and HCT116 cells in 25‐square‐centimetre culture flasks until reaching approximately 50% confluence. Subsequently, the cells were irradiated with a dose of x‐ray and then incubated until they grown to approximately 90% confluence, at which point they were sub‐cultured into new flasks. Then, when they reached approximately 50% confluence again, they were irradiated with a second round of radiation. These steps were repeated a total of sixteen times (1 Gy four times, 2 Gy four times, 4 Gy four times, and 8 Gy four times) over a period of 3 months. All irradiation experiments were performed using a Siemens Primus. H linear accelerator, with a 20 cm × 20 cm field size, a 100 cm source‐to‐skin distance, and a 200 monitor unit (MU)/min dose rate.

### 2.2 Cell transfection

To construct tRF‐16‐7X9PN5D overexpression, knockdown and control models, synthetic analogs and inhibitors of tRF‐16‐7X9PN5D and negative control, oligonucleotides were designed and transfected into SW620 and HCT116 cells via genome editing technology. MKNK1 is small interfering RNA (siRNA), and the corresponding negative control RNA were transfected into both the cell lines in the log growth phase. Lipofectamine 2000 transfection reagent (Invitrogen) was utilized as per the manufacturer's guide. Table S1 enlists the transfection sequences.

### 2.3 tRFs and tiRNAs sequencing

The SW620R and HCT116R radiation‐resistant cell lines and their normal controls, SW620 and HCT116, were used for tRFs and tiRNAs sequencing. The library building and RNA sequencing were performed by KangChen Bio‐tech. The sequencing libraries were quantified via Agilent BioAnalyzer 2100. Using the Illumina NextSeq platform with 50 bp single‐end sequencing, standard small RNA sequencing was carried out. Cytoplasmic and mitochondrial tRNA sequences were acquired and predicted by GtRNAdb and tRNAscan‐SE software, respectively. Predicted intron sequences (if any) were deleted, and an additional 3′‐terminal ‘CCA’ was added to each tRNA to build a mature tRNA library. A precursor tRNA library was generated by adding a flanking genomic sequence of 40 nucleotides at each side of the original tRNA sequence. A corrected *p* < 0.05 was deemed statistically essential. This investigation used RNA sequencing samples with three replicates, each of the radiation‐resistant and normal control groups.

### 2.4 Bioinformatics Analysis

Potential tRF‐16‐7X9PN5D target genes were predicted, and tsRNA‐mRNA network construction was performed by miRanda and TargetScan. Gene Ontology (GO)^36^ and Kyoto Encyclopedia of Genes and Genomes (KEGG)^37^ analyses predicted the biological role and signalling pathways associated with the tRF‐16‐7X9PN5D target genes. The *p*‐values depicted the importance of the GO term or pathway linked with its target genes. The lower the *p*‐value, the more essential the association of the GO term or pathway with its target genes.

### 2.5 Quantitative real‐time polymerase chain reaction (qRT‐PCR)

As per the manufacturer's guide, whole RNA from SW620/R and HCT116/R cells was acquired with the help of a Trizol reagent (Ambion). cDNA was prepared with the help of a reverse transcription system kit (Genepharma). qRT‐PCR was carried out using SYBR Green (Genepharma), and the GAPDH was kept as the endogenous reference on an Agilent Mx3005P fluorescence quantitative PCR instrument to assess the mRNA's relative expression. The tRF's relative expression was measured by the 2^−ΔΔCt^ method. Table S2 presents the details of the primer sequences.

### 2.6 Colony formation assay

Cells (1000/well) in the log phase were propagated in 6‐well plates (NEST). After 2 weeks, cell colonies were rinsed thrice with 1 × PBS, fixed for 30 min by 4% paraformaldehyde (Solarbio), stained for 30 min by 0.5% crystal violet (Solarbio), and counted. Colonies comprising 50 cells were selected to assess the cloning formation rate. Images were acquired using a gel imaging system.

### 2.7 Cell counting kit‐8 (CCK‐8) proliferation assay

With the help of this assay, the CRC cell's proliferative ability was evaluated. Transfected cells (5 × 10^3^/well) were propagated in a 96‐well plate (NEST) at different time points (0, 24, 48, 72 and 96 h). After adherence, 10 μL of CCK‐8 solution (Dojindo) was introduced in each well and kept for 2 h at 37°C. The absorbance was acquired via a microplate reader (Potenov) at 450 nm.

### 2.8 Wound healing assay

This assay was carried out by cultivating cells in a 6‐well plate until 90% confluency, then with the help of a 200 μL pipette tip, same‐width scratches were created, and images were immediately captured by an Olympus microscope. The cells were then incubated for 24 h in a serum‐free medium before capturing images. The scratch width was measured at each time point, and the distance was assessed via Image‐Pro Plus 6.0 software.

### 2.9 Transwell invasion assay

The invasive ability of transfected cells was assessed by the transwell invasion assay. Briefly, cells were collected and resuspended in a serum‐free medium in the matrix gel mixture (BD Biosciences) coated transwell chambers (Corning) were inserted into a 24‐well plate containing 20% serum (Gibco). After 24 h of incubation, the remaining cells on the chamber's upper surface were removed using a cotton swab, and lower surface adhered cells were fixed and stained. The number of invaded cells was evaluated under a microscope and quantified with ImageJ software.

### 2.10 Western blot assay

Cellular proteins were acquired with the help of RIPA lysis buffer (Solarbio). Electrophoresis gel was prepared, and the device was fixed according to the protocol. The buffer and appropriate protein marker were added to the electrophoresis tank, and an equal quantity of protein lysate was introduced in each well in a predetermined order. After transfer, the membrane was sealed with skim milk, then kept at 4°C in primary antibodies (anti‐MKNK1, anti‐FGF10, anti‐EIF4E, anti‐P‐EIF4E and anti‐GAPDH) (Abcam) overnight, followed by secondary antibodies incubation at ambient temperature for 1 h. Finally, the membrane was imaged using the Odyssey system.

### 2.11 Dual‐luciferase reporter gene assay

SW620 and HCT116 cells were propagated in 24‐well plates (Corning) (1 × 10^5^/well) for 24 h before transfection. Luciferase reporter vectors of Wild‐type and mutant *MKNK1* targeting the tRF‐16‐7X9PN5D binding site were constructed. Cells were co‐transfected with the vectors, and tRF‐16‐7X9PN5D mimics via Lipofectamine 2000 reagent. This assay was performed to assess luciferase activity 24 h after transfection.

### 2.12 Statistical measurement

Experimental data were depicted as mean ± standard deviation. Statistical measurement was carried out via SPSS 22.0 (IBM, SPSS) and GraphPad Prism 8.0 (GraphPad) software. All investigations were repeated at least thrice. The intergroup differences were compared by Student's *t*‐test, while multigroup differences were compared by one‐way anova. A *p* < 0.05 was deemed statistically essential.

## 3 RESULTS

### 3.1 Construction of radiation‐resistant SW620/R and HCT116/R cell models

**FIGURE 1:**
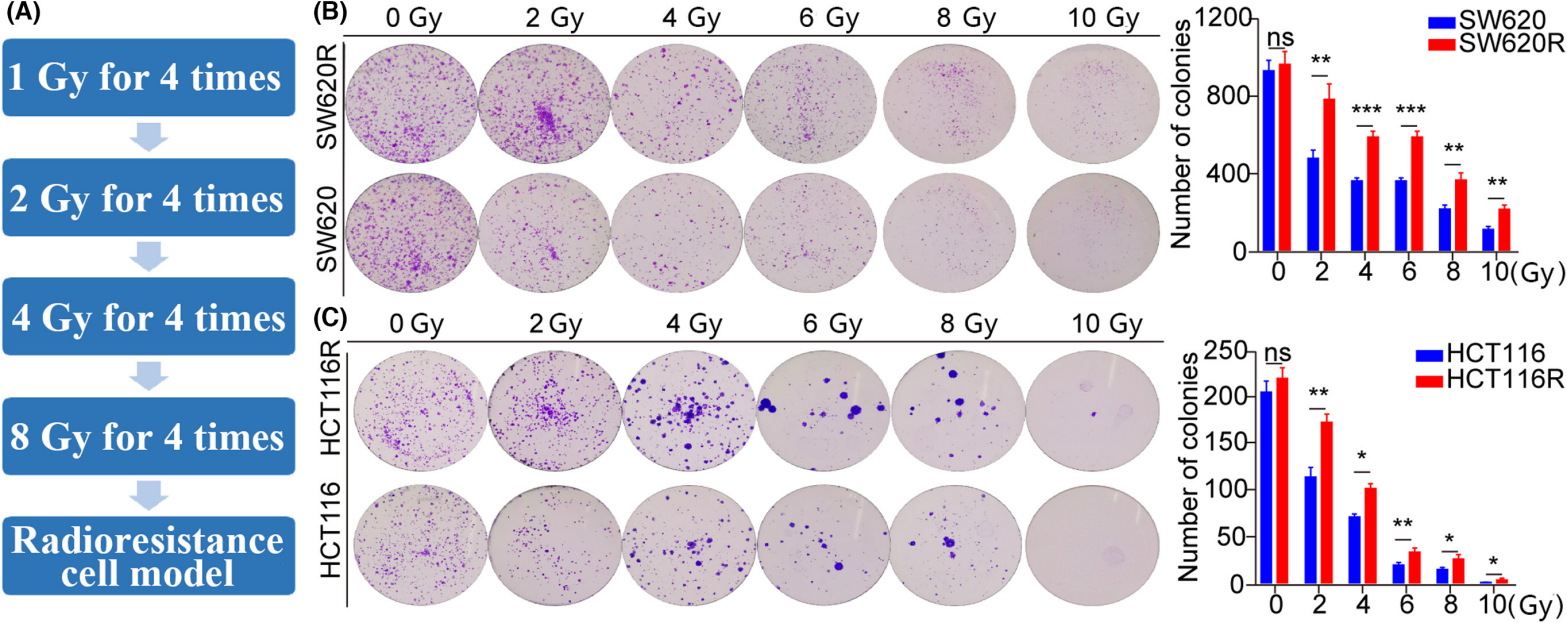
Establishing radiation‐resistant SW620/R and HCT116/R cell models. (A) CRC cell lines were irradiated with different doses (1, 2, 4, 8 Gy) 16 times to establish radioresistance cell model. (B, C) Colony formation assays were used to test the proliferation of CRC SW620/R and HCT116/R cells irradiated with different doses of radiation (2, 4, 6, 8, and 10 Gy). *p < 0.05, **p < 0.01, ***p < 0.001, ns, no significance.

To establish radiation‐resistant cell models, colon cancer cell lines were irradiated with different doses (1, 2, 4, 8 Gy) sixteen times (Figure 1A). The radiation‐resistant (SW620R and HCT116R) and normal control (SW620 and HCT116) groups were irradiated separately with different doses (2, 4, 6, 8, 10 Gy) and then subjected to a colony formation assay, which revealed that in the radiation‐resistant groups, the cell colony formation was markedly higher than the normal control groups, and the difference was statistically essential (Figure 1B). These findings indicate the successful establishment of radiation‐resistant SW620/R and HCT116/R cell models.

### 3.2 High‐throughput sequencing analysis of differentially expressed tsRNAs

**FIGURE 2:**
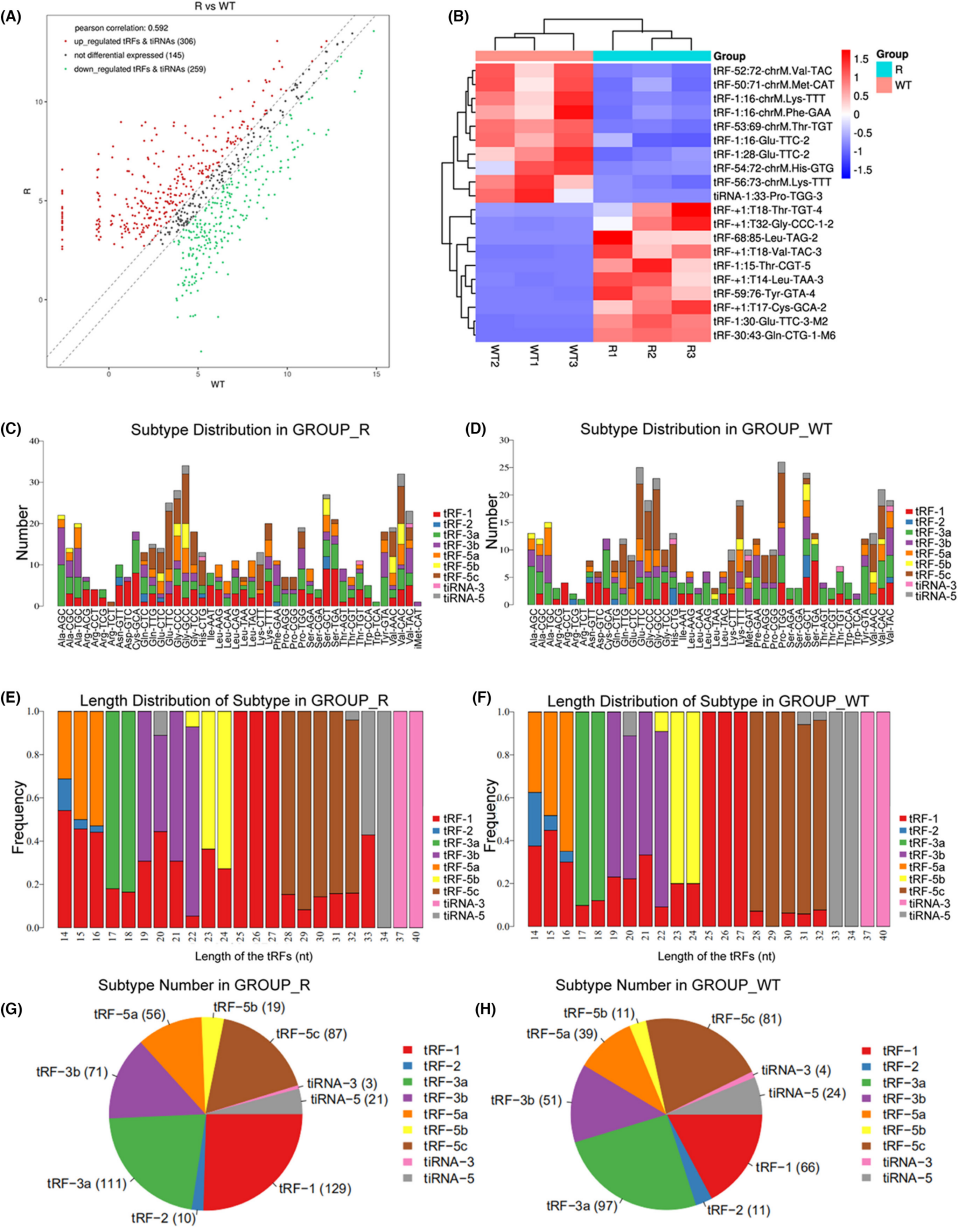
tRF & tiRNA high‐throughput sequencing profile and classification. (A) Scatter plot displays all differentially expressed tsRNAs. (B) Hierarchical cluster heatmap of 20 significantly differentially expressed tsRNAs. (C–F) Subtype distributions and length distributions of different types of tRFs and tiRNAs in CRC SW620/R and HCT116/R cells. (G, H) Pie diagram displaying the ratio of each subtype tsRNAs between radiation‐resistant (SW620R and HCT116R) and normal control (SW620 and HCT116) cells.

**FIGURE 3:**
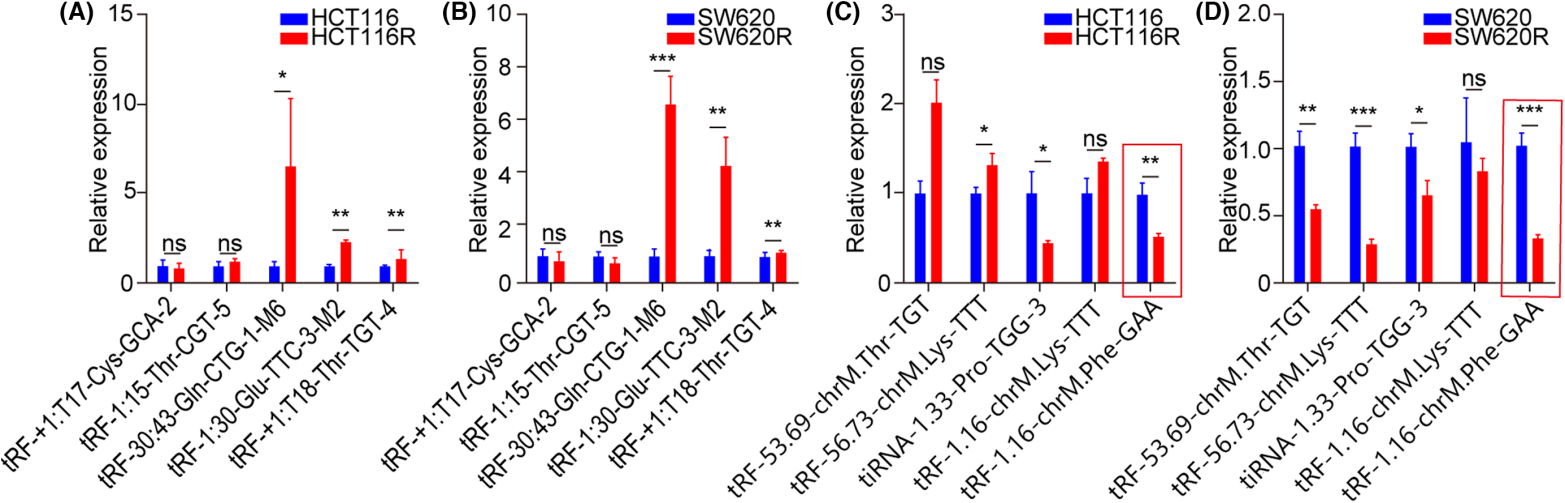
Expression of 10 differentially expressed tsRNAs in SW620/R and HCT116/R cells. (A, B) qPCR validation of 5 tsRNAs with high expression in CRC SW620/R and HCT116/R cell lines. (C, D) qPCR validation of 5 tsRNAs with low expression in CRC SW620/R and HCT116/R cell lines. *p < 0.05, **p < 0.01, ***p < 0.001, ns, no significance.

To elucidate the differential expressed tsRNAs in radiation‐resistant CRC cells, high‐throughput sequencing analysis of tRFs and tiRNAs in SW620/R and HCT116/R cells was performed, revealing substantial differences in tRFs and tiRNAs levels between the radiation‐resistant and wild‐type cells. Three hundred six tsRNAs were upregulated, and 259 were downregulated in the radiation‐resistant cells than in the wild‐type cells (Figure 2A). A clustering heatmap of the top 20 most essential differentially expressed tsRNAs is presented in Figure 2B. Additionally, the sequencing analysis revealed variations in tRFs and tiRNAs between the radiation‐resistant and wild‐type cells, where CRC cells were abundant in tRF‐1, tRF‐3a and tRF‐5c. The levels of tRF‐1, tRF‐3a and tRF‐5a differed significantly between the two cell lines (Figure 2C–H). Subsequently, 10 substantially differentially expressed tsRNAs (5 up‐ and down‐regulated, respectively) were selected for further experiments (Table S3). The expression of these tsRNAs in SW620/R and HCT116/R cells was validated by qRT‐PCR (Figure 3), which revealed that tRF‐30.43‐Gln‐CTG‐1‐M6 and tRF‐1.30‐Glu‐TTC‐3‐M2 were substantially upregulated in both radiation‐resistant cell lines, while tiRNA‐1.33‐Pro‐TGG‐3 and tRF‐1.16‐chrM.Phe‐GAA was markedly downregulated, consistent with the sequencing results. Among them, tRF‐1.16‐chrM.Phe‐GAA (MINTbase_ID: tRF‐16‐7X9PN5D) showed the most significant differential expression; therefore, it was selected for subsequent study.

### 3.3 Inhibition of tRF‐16‐7X9PN5D expression promotes the radioresistance of CRC cells

**FIGURE 4:**
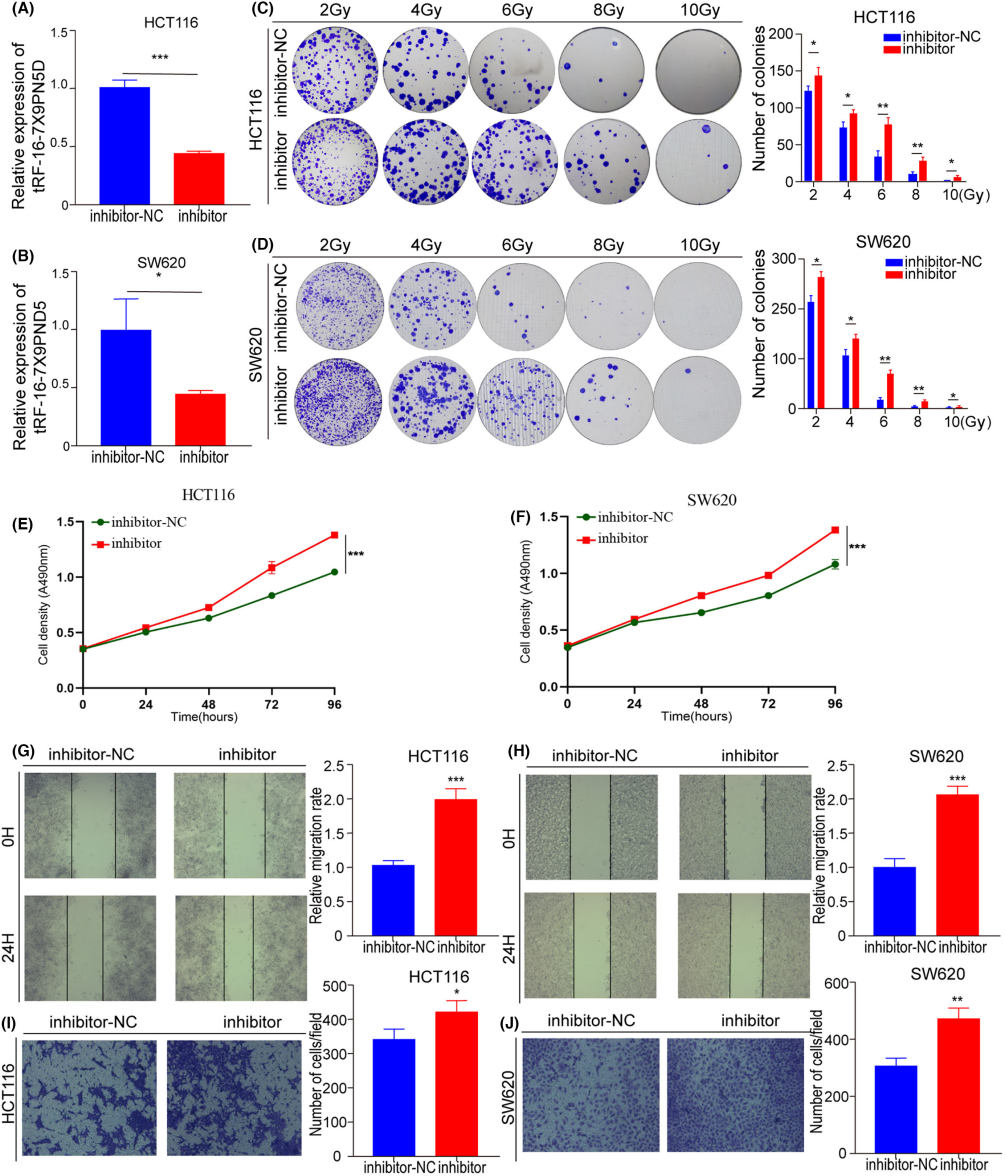
Inhibition of tRF‐16‐7X9PN5D expression promotes radioresistance, proliferation, migration, and invasion of CRC cells. (A, B) Relative expression level of tRF‐16‐7X9PN5D in CRC SW620 and HCT116 cells treated with NC and its inhibitor. (C, D) Colony formation assay indicated that the inhibition of tRF‐16‐7X9PN5D expression promotes CRC SW620 and HCT116 cell proliferation after different doses of irradiation (2, 4, 6, 8, 10 Gy). (E, F) Cell proliferation was analysed using CCK‐8 assays in CRC SW620 and HCT116 cells treated with NC and its inhibitor. (G, H) Cell migration was analysed using wound healing assays in CRC SW620 and HCT116 cells treated with NC and its inhibitor. (I, J) Cell invasion was measured using transwell assays in CRC SW620 and HCT116 cells treated with NC and its inhibitor. *p < 0.05, **p < 0.01, ***p < 0.001, ns, no significance.

To further explore the correlation between tRF‐16‐7X9PN5D and radioresistant CRC cells, tRF‐16‐7X9PN5D inhibitors were transfected into HCT116 and SW620 cells separately. These were designated as the inhibition group, while the wild‐type (HCT116 and SW620) cells without transfection were called the control group. The qRT‐PCR detected tRF‐16‐7X9PN5D expression in both groups. It was observed that tRF‐16‐7X9PN5D expression in the inhibition group was markedly reduced, and the difference was statistically important (Figure 4A,B). Subsequently, a colony formation assay was performed on both groups after different doses of irradiation (2, 4, 6, 8, 10 Gy), which indicated that the inhibition group's cell cloning formation ability was statistically notably higher than the control group (Figure 4C,D), suggesting that inhibiting tRF‐16‐7X9PN5D expression can promote the radioresistance of CRC cells.

### 3.4 Inhibition of tRF‐16‐7X9PN5D expression promotes proliferation, migration and invasion of CRC cells

The effects of tRF‐16‐7X9PN5D on CRC cell function were determined. CCK‐8 assay indicated that the cell density of the tRF‐16‐7X9PN5D inhibition group was notably higher than the control group, and the results were statistically important (Figures 4E,F). This indicates that inhibiting tRF‐16‐7X9PN5D can promote CRC cell proliferation. Furthermore, according to the wound healing assay (Figures 4G,H), the migration distance of the tRF‐16‐7X9PN5D inhibition group was substantially elevated than the control group after 24 h of cultivation as well as the migration rate. This indicates that tRF‐16‐7X9PN5D inhibition stimulates CRC cell migration. The cell invasion assay results (Figures 4I,J) showed that the cell invasion number of the tRF‐16‐7X9PN5D inhibition group was substantially increased than the control group, suggesting that tRF‐16‐7X9PN5D suppression stimulates the invasion of CRC cells. Altogether, it was discovered that inhibiting the expression of tRF‐16‐7X9PN5D can promote CRC cells' ability to proliferate, migrate, and invade.

### 3.5 MKNK1 is a direct target gene of tRF‐16‐7X9PN5D

**FIGURE 5:**
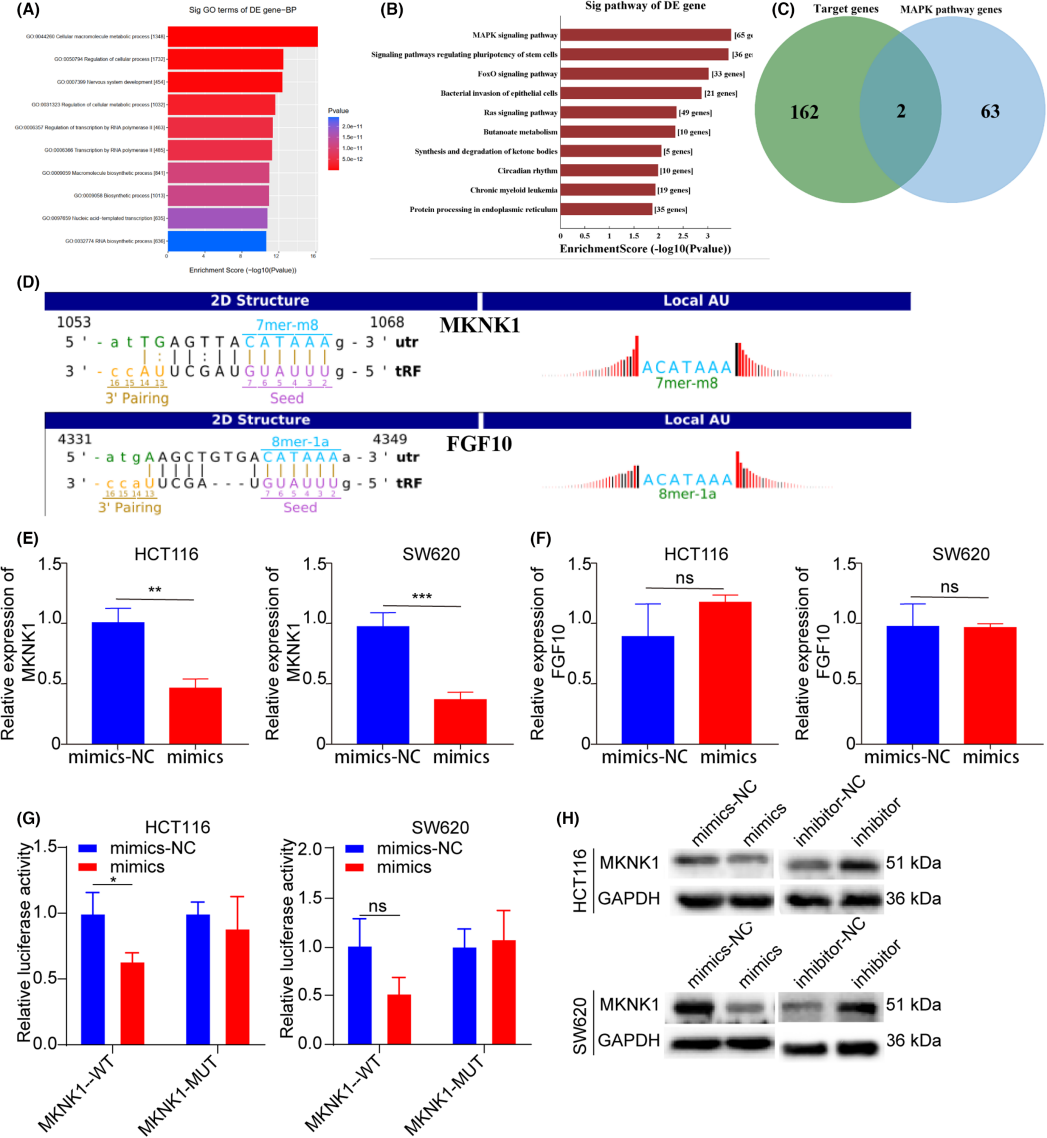
MKNK1 is tRF‐16‐7X9PN5D direct target gene. (A) GO analysis revealed that the target genes of tRF‐16‐7X9PN5D were most closely related to cellular macromolecule metabolic processes. (B) KEGG analysis revealed that the target genes of tRF‐16‐7X9PN5D was significantly correlated with MAPK pathway genes. (C) Venn diagram presenting the target genes of tRF‐16‐7X9PN5D and MAPK signalling pathway. (D) The prediction of binding site between tRF‐16‐7X9PN5D 3′ UTR and *MKNK1* and *FGF10*. (E, F) Relative expression level of *MKNK1* and *FGF10* in CRC SW620 and HCT116 cells treated with NC and tRF‐16‐7X9PN5D mimics. (G) The *MKNK1* WT or MUT reporter plasmid was co‐transfected with RNA oligonucleotides (NC, mimics) into HCT116 and SW620 cells. (H) Effects of tRF‐16‐7X9PN5D on the expression of MKNK1 proteins in HCT116 and SW620 cells. *p < 0.05, **p < 0.01, ***p < 0.001, ns, no significance.

**FIGURE 6:**
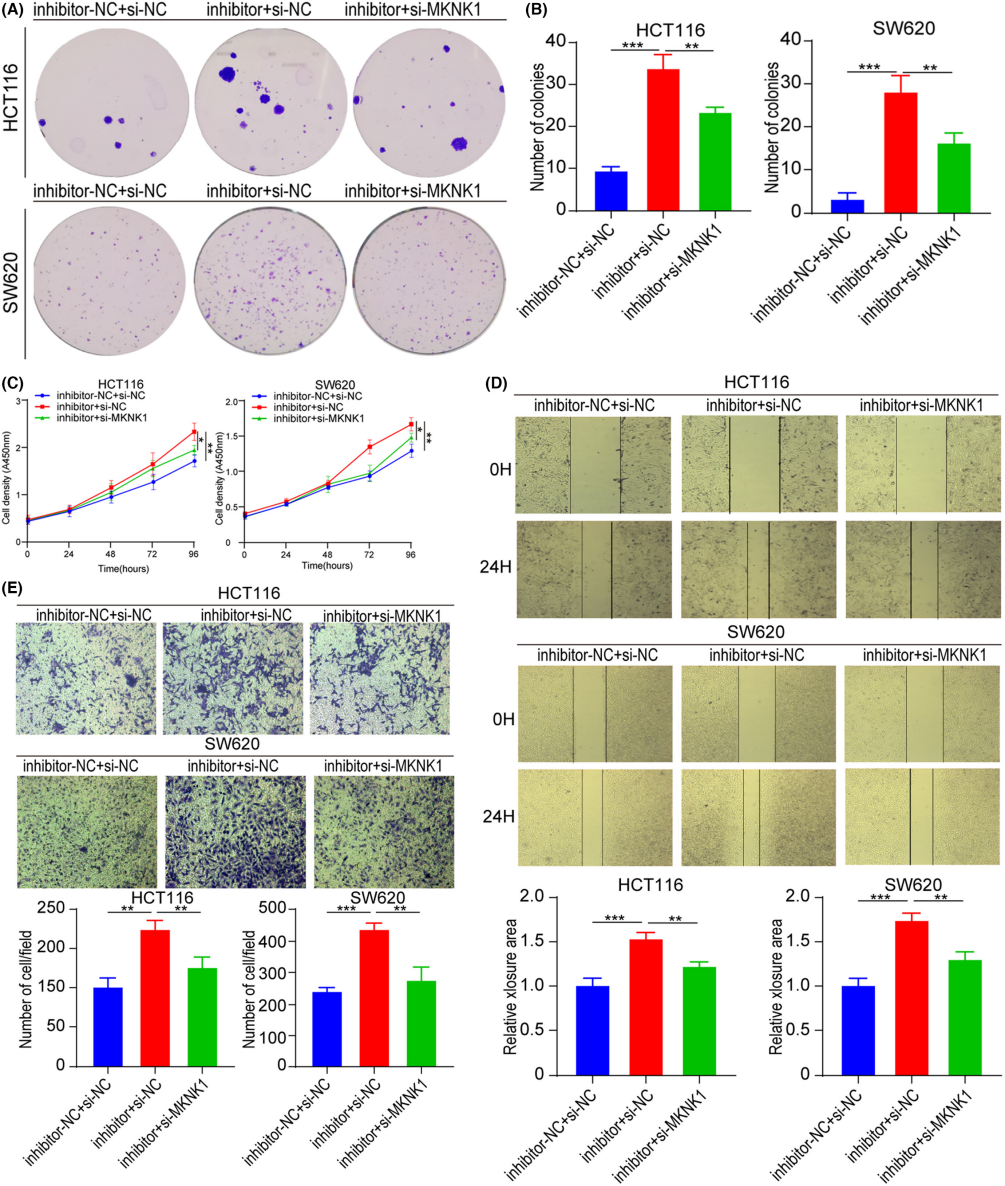
tRF‐16‐7X9PN5D promotes CRC cell's ability of radioresistance, proliferation, migration, and invasion by targeting *MKNK1*. (A, B) Rescue assays showed that co‐transfection of tRF‐16‐7X9PN5D inhibitor and si‐MKNK1 suppressed the promoting ability of tRF‐16‐7X9PN5D inhibitor on cell radioresistance of CRC HCT116 and SW620 cells after 6Gy doses of irradiation. *p < 0.05, **p < 0.01, ***p < 0.001, ns, no significance. (C–E) Rescue assays showed that co‐transfection of tRF‐16‐7X9PN5D inhibitor and si‐MKNK1 suppressed the promoting ability of tRF‐16‐7X9PN5D inhibitor on cell proliferation (C), migration (D), and invasion (E) of CRC HCT116 and SW620 cells. *p < 0.05, **p < 0.01, ***p < 0.001, ns, no significance.

GO, and KEGG assessments predicted the biological activity and linked signalling pathways of tRF‐16‐7X9PN5D (Figure 5A,B). According to the GO analysis, the target tRF‐16‐7X9PN5D genes were most substantially related to cellular macromolecule metabolic processes. Whereas KEGG indicated that the target tRF‐16‐7X9PN5D genes were markedly enriched in the MAPK signalling pathway. Furthermore, the downstream direct tRF‐16‐7X9PN5D targets were explored by miRanda and TargetScan to predict 162 potential target genes of tRF‐16‐7X9PN5D (Table S4). Taking the intersection of the 162 genes and the target genes of the MAPK signalling pathway (Tables S4 and S5), *MKNK1* and *FGF10* were identified as the common target genes (Figure 5C,D). The relationship between tRF‐16‐7X9PN5D and *MKNK1* and *FGF10* was verified through experiments. HCT116 and SW620 cells were transfected with tRF‐16‐7X9PN5D mimics and negative controls as the overexpression and control groups. qRT‐PCR revealed that *MKNK1* expression was markedly lower in the overexpression group than in the control group, while no marked variation was observed in the FGF10 levels between the two groups (Figure 5E,F). According to the dual luciferase assay, tRF‐16‐7X9PN5D overexpression substantially inhibited wild‐type *MKNK1* activity but not that of mutant *MKNK1* (Figure 5G). The western blotting indicated that tRF‐16‐7X9PN5D overexpression could suppress *MKNK1* while inhibiting tRF‐16‐7X9PN5D has the opposite effect (Figure 6H). In conclusion, *MKNK1* is a direct target gene of tRF‐16‐7X9PN5D.

### 3.6 tRF‐16‐7X9PN5D regulates radioresistance in CRC cells by directly targeting *MKNK1*

To further investigate the molecular mechanism by which tRF‐16‐7X9PN5D induces radioresistance, both the cell lines were categorized into three groups. The first group was transfected with negative control oligonucleotides and siRNA, the second group was transfected with tRF‐16‐7X9PN5D inhibitor and negative control siRNA, and the third group was transfected with both tRF‐16‐7X9PN5D inhibitor and si‐MKNK1. After 6 Gy irradiation, a colony formation assay was carried out to verify the results, which indicated that the cell clone formation rate in the second group was substantially elevated than the first group, while its rate in the third group was notably lower than the second group (Figure 6A,B), suggesting that tRF‐16‐7X9PN5D stimulates radioresistance in CRC cells by directly targeting *MKNK1*.

### 3.7 tRF‐16‐7X9PN5D directly targets *MKNK1* to regulate the proliferation, migration and invasion of CRC cells

The underlying molecular mechanisms of modulation of cellular activity by tRF‐16‐7X9PN5D were further assessed by rescue experiments. CCK‐8 experiment also revealed that the cell density in the third group was markedly lower than the second group, indicating that *MKNK1* can partially reverse the promoting effect of tRF‐16‐7X9PN5D inhibition on CRC cell proliferation (Figure 6C). The wound healing assay indicated that the migration distance of the second group was markedly elevated than the first group after 24 h of culture, while that of the third group was notably shortened than the second group, suggesting that *MKNK1* can reverse the promoting effect of inhibiting tRF‐16‐7X9PN5D expression on the migration of CRC cells (Figure 6D). According to the cell invasion experiment, the number of migrating cells in the second group was markedly enhanced than the first and third groups, indicating that *MKNK1* alters the promoting effect of inhibiting tRF‐16‐7X9PN5D expression on the invasion of CRC cells (Figure 6E). Overall, tRF‐16‐7X9PN5D directly targets *MKNK1* to stimulate CRC cell's ability to proliferate, migrate and invade.

### 3.8 tRF‐16‐7X9PN5D and MKNK‐EIF4E axis

**FIGURE 7:**
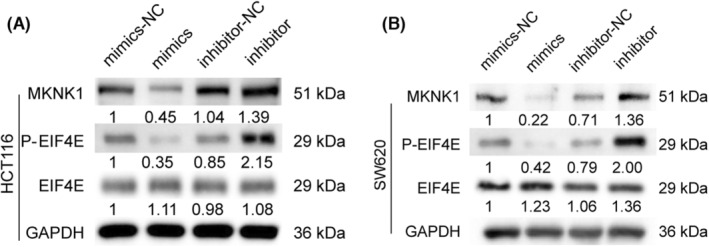
tRF‐16‐7X9PN5D regulates the expression of MKNK1 and P‐EIF4E. (A, B) Effects of tRF‐16‐7X9PN5D on the expression of MKNK1, EIF4E and P‐EIF4E proteins in HCT116 and SW620 cells.

Previous studies have revealed that MKNK1 can phosphorylate eukaryotic initiation factor 4E (eIF4E) and co‐regulate various cellular processes,^24^ and essentially regulate the progression of different cancers.^38^ Therefore, preliminary verification was conducted by protein immunoblotting, which indicated that after tRF‐16‐7X9PN5D mimics transfection in HCT116 and SW620 cells, MKNK1 and P‐EIF4E were markedly reduced than the control group, while the EIF4E expression remained unaltered. Meanwhile, after tRF‐16‐7X9PN5D inhibitor transfection, MKNK1 and P‐EIF4E increased substantially, while the EIF4E expression remained unaltered (Figure 7A,B). Therefore, it can be concluded that tRF‐16‐7X9PN5D can regulate the phosphorylation of eIF4E through MKNK1, thereby exerting its biological functions.

## 4 DISCUSSION

This investigation utilized RNA sequencing technology to elucidate the relationship between tsRNAs and radiation resistance in CRC. It was indicated that the expression of tsRNAs in normal and radiation‐resistant CRC cell lines had substantial differences. Among them, 306 tsRNAs were upregulated, and 259 were downregulated in radiation‐resistant CRC cells than in the wild‐type cells. Ten tsRNAs with marked differences were selected for qPCR validation and indicated that a tRF‐5a fragment tRF‐16‐7X9PN5D was substantially downregulated in both radiation‐resistant CRC cell lines, consistent with the sequencing results. Transwell invasion, colony formation, wound healing and CCK‐8 assays confirmed that inhibiting the expression of tRF‐16‐7X9PN5D significantly stimulated CRC cells' ability to proliferate, migrate, and invade. Furthermore, it was also discovered that *MKNK1* was a direct potential tRF‐16‐7X9PN5D target gene. Subsequent cell experiments showed that tRF‐16‐7X9PN5D might promote CRC cells' ability to proliferate, migrate, invade, and have radiation resistance by regulating *MKNK1*. Finally, the relationship between tRF‐16‐7X9PN5D and the MKNK‐eIF4E axis was verified, and it was found that tRF‐16‐7X9PN5D overexpression inhibits the levels of MKNK1 and P‐EIF4E, while tRF‐16‐7X9PN5D expression inhibition promoted their levels. These findings suggest that tRF‐16‐7X9PN5D and the tRF‐16‐7X9PN5D‐MNNK1‐eIF4E axis may serve as a new pathway for regulating the sensitivity of CRC to radiation therapy and provide new ideas for improving radiation resistance in CRC.

Much research shows that tsRNAs essentially regulate CRC. For example, the ectopic tRF/miR‐1280 expression suppresses cell proliferation and tumour growth,^39^ tRF‐20‐M0NK5Y93 inhibits cell's ability to migrate, invade, and metastasize,^40^ 5'tiRNA‐His‐GTG upregulation stimulates tumour cell proliferation and transplanted tumour's formation and growth, while its inhibition markedly reduces tumour cell colony formation and induces apoptosis,^41^ and tRF3008A overexpression inhibits cell migration, growth, apoptosis and invasion.^18^ Studies also show that tsRNAs can serve as diagnostic and CRC prognostic indices. For example, Chen et al. showed that the ROC curve assessment of tRF‐Phe‐GAA‐031 expression between tumour and paracancerous tissue samples had an AUC of 0.7554, while tRF‐VAL‐TCA‐002 had an AUC of 0.7313.^42^ Patients with high tRF‐Phe‐GAA‐031 and tRF‐VAL‐TCA‐002 levels had substandard overall survival (OS).^42^ In addition, a study found that CRC patients with low expression of tRF3008A had significantly shortened disease‐free survival (DFS),^18^ and those with high expression of 5’‐tiRNA‐Pro^TGG^ had significantly shortened DFS and OS.^43^ However, there are currently no reports on the association of tsRNAs in modulating cancer cell's radiation sensitivity.

The MAPK is an important signal transduction pathway with three main branches: ERK, JNK and p38.^44^ Studies suggest that its activity is essentially important for the activation and regulation of *MKNK1*. Specifically, ERK1/2 and p38MAPK can directly phosphorylate and activate MKNK1, promoting MKNK1‐mediated eIF4E phosphorylation, protein synthesis, tumour cell proliferation and metastasis.^25^, ^45^, ^46^ There are also studies indicating a close relationship between the MAPK signalling pathway and tumour radiotherapy. Kumar, et al.'s study, showed that gamma radiation stimulates endothelial cell apoptosis by p38 MAPK activation, while vascular endothelial growth factor protects these cells from gamma radiation via the PI3K‐Akt‐Bcl‐2 signalling pathway.^47^ Therefore, the MAPK signalling pathway and *MKNK1* are closely linked with the incidence and development of tumours. This investigation indicated that tRF‐16‐7X9PN5D could affect CRC cells' radiation resistance by targeting *MKNK1*, which has important guiding significance for tumour radiotherapy.

*MKNK1* is a serine/threonine kinase and a member of the MAPK subfamily, essential for cancer development and progression, and has potential clinical applications. Berger et al. indicated that *MKNK1* polymorphism rs8602 could furnish a predictive index for the efficacy of FOLFIRI with bevacizumab therapy in patients with wild‐type KRAS metastatic CRC receiving first‐line treatment.^31^ AA genotype carriers have a poorer treatment response rate and progression‐free survival (PFS).^31^ Knight et al. revealed that *MKNK1/2* loss could inhibit the KRAS‐mutant proliferation of CRC cells because of rapamycin and increase drug therapy sensitivity.^48^ According to Astanehe et al., *MKNK1* could mediate targeted drug resistance in HER2‐positive breast cancer, and its expression downregulation by siRNA can increase the cancer cells' sensitivity to trastuzumab.^49^ Bell et al. indicated that *MKNK1* expression is notably increased in Glioblastoma multiforme compared to Grades II and III gliomas, and its upregulation indicates a substandard prognosis.^33^ Additionally, In HER2‐overexpressing breast cancer cells, *MNK1/2* inhibition can weaken colony‐forming ability, suggesting that this gene stimulates cancer cell growth.^32^ In epithelial ovarian cancer (EOC) cells, *MKNK1* can promote proliferation, and its elevated expression is notably linked with later‐stage and positive lymph node metastasis, making it an independent prognostic factor for OS in EOC patients.^50^

MKNK1 phosphorylates eIF4E to form the MKNK‐eIF4E axis,^24^, ^51^, ^52^ which is a widely studied biological pathway that participates in many cellular processes, including regulating transcription, cell proliferation, translation and survival.^53^ In some cases, especially in the progression of certain cancers, abnormal activation of this axis causes excessive eIF4E phosphorylation, thereby promoting growth, proliferation and cancer cell metastasis.^54^ Recently it has been suggested that the MKNK‐eIF4E axis is critically involved in various cancers.^55^ For example, Galeterone and VNPT55 inhibit the migration and invasion of prostate cancer cells by disrupting the MKNK‐eIF4E axis.^38^ Inhibiting this axis disrupts melanoma phenotype switching and enhances anti‐tumour immune responses.^56^ Knocking out *MKNK1* can alleviate phosphorylated eIF4E and tumour formation in glioblastoma U87MG cells.^57^ It has also been indicated that activated MKNK‐eIF4E axis levels are closely linked with the malignancy, treatment response and prognosis of certain cancers.^58^ Therefore, the MKNK‐eIF4E axis is also considered an efficient therapeutic target, and the development and application of its inhibitors in cancer treatment have important clinical prospects.^46^, ^57^ Some drugs have been developed and entered clinical trials, including MKNK kinase and eIF4E inhibitors.^53^, ^59^, ^60^ This research has found that tRF‐16‐7X9PN5D can affect the invasion, growth, migration and radiation sensitivity of CRC cells via *MKNK1* and has a critical regulatory effect on the MKNK‐eIF4E axis, suggesting that the tRF‐16‐7X9PN5D‐MKNK1‐eIF4E axis may be markedly associated with CRC progression and radiation resistance.

The limitations include: (1) only two common colon cancer cell lines were selected as radiation‐resistant and wild‐type cell lines for sequencing and as control, respectively, and the sequencing results may not have covered all up‐ and down‐regulated tsRNAs. (2) the relationship between tRF‐16‐7X9PN5D and downstream MAPK signalling pathway molecules was tentatively explored. (3) it was the first study to indicate that tRF‐16‐7X9PN5D regulates colon cancer radiation resistance by targeting *MKNK1* in vitro; however, in vivo research is needed to confirm these findings.

## 5 CONCLUSION

In summary, this article provides sufficient evidence to confirm that downregulation of tRF‐16‐7X9PN5D promotes radiation resistance of CRC cells. Furthermore, our study identified *MKNK1* as a direct tRF‐16‐7X9PN5D target gene, and tRF‐16‐7X9PN5D regulates CRC cell's ability to grow, migrate, invade, and obtain radiation resistance via *MKNK1*. Moreover, we found that tRF‐16‐7X9PN5D can regulate the phosphorylation of eIF4E through *MKNK1*, thereby exerting its biological functions. Therefore, we concluded that the tRF‐16‐7X9PN5D‐MNNK1‐eIF4E axis may serve as a novel pathway for regulating the radiosensitivity of CRC. These findings provide new potential regulatory targets for CRC radiotherapy and offer new explanations for the occurrence of CRC radiation resistance.

## AUTHOR CONTRIBUTIONS

**Tianyi Huang:** Data curation (equal); writing – original draft (lead). **Chujia Chen:** Formal analysis (lead); writing – original draft (supporting). **Juan Du:** Investigation (lead); software (equal). **Zhen Zheng:** Data curation (equal); software (equal). **Shuang Ye:** Formal analysis (supporting); project administration (lead). **Shuai Fang:** Resources (lead); supervision (equal); writing – review and editing (supporting). **Kaitai Liu:** Conceptualization (lead); supervision (equal); writing – review and editing (lead).

## FUNDING INFORMATION

This study was supported by grants from the Project of NINGBO Leading Medical & Health Discipline (No. 2022‐F01); the Ningbo Natural Science Foundation (No. 2021J292); the Medical Science and Technology Project of Zhejiang Provincial Health Commission (No. 2023KY243); the Natural Science Foundation of Zhejiang Province (No. LY21H160013).

## CONFLICT OF INTEREST STATEMENT

The authors declare that they have no competing interests.

## Supporting information


Table S1.

Table S4.

## Data Availability

The data that support the findings of this study are available from the corresponding author upon reasonable request.
