# Supplementary material for: A tRF‐5a fragment that regulates radiation resistance of colorectal cancer cells by targeting MKNK1
Source: J Cell Mol Med. 2023 Oct 21;27(24):4021–33. doi: 10.1111/jcmm.17982 (PMC10747413; doi:10.1111/jcmm.17982)
Supplement: Supplementary file 1 — Table S1. [file JCMM-27-4021-s002.docx]

**Table S1** RNA oligonucleotide sequences.

| **Primer** | **Forward primer (5’ to 3’)** | **Reverse primer (5’ to 3’)** |
| --- | --- | --- |
| MKNK1(for qPCR) | AATTGCTTGGAGAGGGAGCC | TTCACTGGAGATGTGTGCCC |
| GAPDH (for qPCR) | ACCCACTCCTCCACCTTTGAC | TGTTGCTGTAGCCAAATTCGTT |
| FGF10(for qPCR) | ACTGGCAGCATAATGGGAGG | GCAGAGGTGTTTTTCCTTCGT |

**Table S2** Primer sequences.

| **RNA oligos** | **Sequences** |
| --- | --- |
| siRNA-NC | Sense: 5’- UUCUCCGAACGUGUCACGUTT -3’  Anti-sense: 5’- ACGUGACACGUUCGGAGAATT -3’ |
| si-MKNK1 | Sense: 5’- GAAGAUAUGUACAAGCUGACCTT -3’  Anti-sense: 5’- UCAGCUUGUACAUAUCUUCAATT -3’ |

**Table S3** 10 differentially expressed tiRNAs

| **tRF_ID** | **Type** | **log2FC** | **Fold_Change** | **p_value** |
| --- | --- | --- | --- | --- |
| tRF-+1:T17-Cys-GCA-2 | tRF-1 | 6.946921993 | 123.3763442 | 7.58669E-23 |
| tRF-1:15-Thr-CGT-5 | tRF-5a | 6.917603499 | 120.8943909 | 1.82757E-24 |
| tRF-30:43-Gln-CTG-1-M6 | tRF-2 | 6.047459092 | 66.14036455 | 5.89054E-21 |
| tRF-1:30-Glu-TTC-3-M2 | tRF-5c | 5.668741546 | 50.86994206 | 3.88147E-21 |
| tRF-+1:T18-Thr-TGT-4 | tRF-1 | 5.30401452 | 39.50640102 | 3.93902E-11 |
| tRF-53:69-chrM.Thr-TGT | tRF-3a | -4.744268847 | 0.037310646 | 4.15707E-19 |
| tRF-56:73-chrM.Lys-TTT | tRF-3a | -4.017504088 | 0.061746275 | 5.37845E-14 |
| tiRNA-1:33-Pro-TGG-3 | tiRNA-5 | -3.953574608 | 0.064543937 | 1.02E-06 |
| tRF-1:16-chrM.Lys-TTT | tRF-5a | -3.849130182 | 0.069389915 | 7.03223E-14 |
| tRF-16-7X9PN5D | tRF-5a | -3.729499176 | 0.075389156 | 7.14974E-11 |
